# Editorial: Control of plant diseases with deep learning

**DOI:** 10.3389/fpls.2024.1354927

**Published:** 2024-01-11

**Authors:** Guoxiong Zhou, Aibin Chen, Weiwei Cai, Liang Gong, Xiaoyulong Chen, Yanfeng Wang

**Affiliations:** ^1^College of Computer & Information Engineering, Central South University Forestry and Technology, Changsha, China; ^2^Missouri University of Science & Technology, Jiangnan University, Wuxi, China; ^3^MoE Key Lab of Artificial Intelligence, AI Institute, Shanghai Jiao Tong University, Shanghai, China; ^4^Text Computing & Cognitive Intelligence Engineering Research Center of National Education Ministry, College of Computer Science and Technology, Guizhou University, Guiyang, China; ^5^College of Systems Engineering, National University of Defense Technology, Changsha, China

**Keywords:** deep learning, plant diseases, diseases identification, recognition model, smart agriculture systems

Plant disease can seriously affect the growth and development of plants, resulting in reduced production and even death. Disease control can assist in plant breeding and cultivation, ensuring crop yield and quality. But traditional disease control methods are increasingly difficult to meet modern time, space and cost requirements. Deep learning technology has developed rapidly in recent years; it can improve the efficiency of plant disease diagnosis and detection, and greatly reduce the risk of human intervention and misjudgment. However, challenges such as data acquisition, environmental interference, and model deployment complexity hinder the application of deep learning in plant disease control. Therefore, there is an urgent need for disease feature optimization algorithms to protect current large-scale agricultural production.

This Research Topic provides a platform for sharing the latest research results of deep learning in control of plant disease. Advances in deep learning techniques in this field mainly concern the application of advanced techniques to the adaptation of plant disease characteristics (Sekmen et al., 2021). The focus on the progress of deep learning technology in this field mainly involves the application of advanced technology in plant disease feature adaptation. This Research Topic aims to explore the latest research and application of deep learning technology in plant disease identification and control, focusing on: 1) identification techniques for early plant disease, 2) prediction and prevention of the spread of plant disease, 3) new methods for collecting multi-source plant disease image data, 4) quantitative evaluation of plant damage, 5) real-time application deployment based on embedded devices and edge wearable devices, 6) adaptive image processing methods for different application environments.

A study by Yang et al. explored a new maize weed recognition model, SE-VGG16, and applied the SE-VGG16 model to weed detection of other species to build a weed detection model with the ability to generalize weed control in agricultural production processes. SE-VGG16 model is based on VGG16, adding SE attention mechanism. The model implements automatic focusing and allocates limited information processing resources to important parts. It has the characteristics of high efficiency, stability and high detection accuracy.

Gole et al. focused on lightweight visual converter networks in a study of automatic plant disease recognition. This study mainly introduces two tasks: one is to replace a multilayer perceptron (MLP) with the initial module and reconstruct the transformer Inception module to reduce the computational complexity of the vision transformer (ViT) model. In addition, the proposed new network is trained and tested on the PlantVillage dataset and the field maize image dataset, which proves its applicability in real scenarios.

Further, Xu et al. studied a cotton moth infestation monitoring method. In this study, five different models of cotton aphid infestation identification were constructed, trained and tested, among which YOLOv5 model had the best performance. The real-time identification system established in this study can provide a faster and more convenient technical means for the monitoring of cotton aphid infection.

In another article, Choi et al. focused on identifying and tracking the behavior, movement, size, and habits of different species of insects by building a 3D monitoring system. While traditional 2D monitoring systems are static and ignore Z-axis depth dimensions, 3D systems are able to track insect movement more comprehensively, providing valuable depth dimension insights.

Another study by Cai et al., explored a lightweight CNN model, FCA-EfficientNet, to achieve accurate identification of multiple maize disease. The work introduced autofocus modules and FCA modules to filter out background noise, allowing the network to focus on disease areas. It also makes the model more lightweight by removing redundant network structures.

In addition, Nan et al. studied the classification of maize disease from another angle. They focused on a deep convolutional neural network with RGB-D images. Among them, a variety of improved data preprocessing methods improved the accuracy and speed of maize diseases image recognition.

A study by Zhang et al. explored the potential of applying Generative Adversarial Networks (GANs) to the diagnosis of cotton fusarium wilt. This study designed a model based on small sample learning and proposed an innovative cotton verticillium wilt diagnosis system. The convolutional neural network is used as feature extractor, and the trained GANs model is used for sample enhancement to improve the classification accuracy.

In summary, these findings highlight the significant advances that deep learning technology has made in identifying and controlling plant disease in the information age. In the future, these models can be deployed in smart agriculture systems to monitor and identify plant disease in real time, so that appropriate control measures can be taken in a timely manner. As deep learning technology continues to evolve and improve, we can expect these models to play an increasingly important role in plant disease control, providing more effective support for agricultural production.

## Author contributions

GZ: Writing – original draft, Writing – review & editing. AC: Conceptualization, Writing – review & editing. WC: Investigation, Writing – review & editing. LG: Supervision, Writing – review & editing. XC: Validation, Writing – review & editing. YW: Supervision, Writing – review & editing.
